# MitoQ improves mitochondrial dysfunction in heart failure induced by pressure overload

**DOI:** 10.1016/j.freeradbiomed.2018.01.012

**Published:** 2018-03

**Authors:** Rogério Faustino Ribeiro Junior, Erinne Rose Dabkowski, Kadambari Chandra Shekar, Kelly A. O´Connell, Peter A. Hecker, Michael P. Murphy

**Affiliations:** a Division of Cardiology, Department of Medicine, University of Maryland, Baltimore, MD, USA; b Department of Integrative Biology and Physiology, University of Minnesota, Minneapolis, MN, USA; c Department of Physiological Sciences, Federal University of Espirito Santo, Vitoria, ES, Brazil; d Medical Research Council Mitochondrial Biology Unit, Cambridge BioMedical Campus, Cambridge, UK

**Keywords:** Heart failure, Mitochondrial dysfunction, MitoQ, Interfibrillar mitochondria, Subsarcolemmal mitochondria, Reactive oxygen species

## Abstract

Heart failure remains a major public-health problem with an increase in the number of patients worsening from this disease. Despite current medical therapy, the condition still has a poor prognosis. Heart failure is complex but mitochondrial dysfunction seems to be an important target to improve cardiac function directly.

Our goal was to analyze the effects of MitoQ (100 µM in drinking water) on the development and progression of heart failure induced by pressure overload after 14 weeks. The main findings are that pressure overload-induced heart failure in rats decreased cardiac function *in vivo* that was not altered by MitoQ. However, we observed a reduction in right ventricular hypertrophy and lung congestion in heart failure animals treated with MitoQ. Heart failure also decreased total mitochondrial protein content, mitochondrial membrane potential in the intermyofibrillar mitochondria. MitoQ restored membrane potential in IFM but did not restore mitochondrial protein content. These alterations are associated with the impairment of basal and stimulated mitochondrial respiration in IFM and SSM induced by heart failure. Moreover, MitoQ restored mitochondrial respiration in heart failure induced by pressure overload. We also detected higher levels of hydrogen peroxide production in heart failure and MitoQ restored the increase in ROS production. MitoQ was also able to improve mitochondrial calcium retention capacity, mainly in the SSM whereas in the IFM we observed a small alteration. In summary, MitoQ improves mitochondrial dysfunction in heart failure induced by pressure overload, by decreasing hydrogen peroxide formation, improving mitochondrial respiration and improving mPTP opening.

## Graphical abstract

**Figure:**
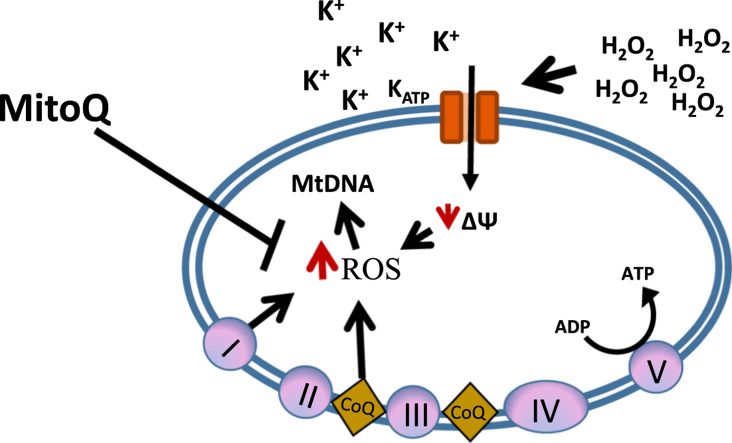

## 1 Introduction

Despite diagnosis and treatment, heart failure remains a major clinical problem and a huge economic burden on the health care system. The incidence and costs are projected to double in the next twenty years. The disease is highly prevalent in elderly patients and is associated with high mortality rates within five years of diagnosis despite current optimal medical therapy. Current medical therapy can prevent new onset and slow the progression once heart failure is established but prognosis is still poor even for most favorable treated patients, and new therapeutic approaches are needed. Heart failure rehospitalizations rates remain high (35%) within 90 days after discharge and this event has not changed over the last 15 years [1], [2], [3], [4], [5].

Commonly prescribed therapies although beneficial in improving some symptoms often do not approach the underlying causes of progressive left ventricular dysfunction presented in heart failure [6].

Left ventricular dysfunction is accompanied by derangements in myocardial fuel metabolism and bioenergetics that contribute to the development and progression of the disease. The energy supply must match the energy demand in the adult heart due to its high requirement of energy to sustain contractile function. In the adult heart, almost all energy production comes from mitochondrial oxidative phosphorylation continually generated by oxidation of carbon fuels [7], [8], [9], [10], [11]. Growing evidence provides information that alterations in mitochondrial ATP production are causally linked to the development of heart failure. Notably, it has been shown that alterations in fatty acid oxidation and oxidative phosphorylation can cause cardiomyopathy in humans and decreased levels of phosphocreatine accelerates the decline during the transition from left ventricular hypertrophy to heart failure [12], [13], [14], [15].

In addition, studies in animals and humans have revealed reprogramming of myocardial fuel utilization in the failing heart: a shift from the fatty acid oxidation (FAO) to increased reliance on glucose utilization. The mechanisms through which the failing heart shifts substrate utilization are still poorly understood.

Moreover, mitochondria are recognized as the main source of reactive oxygen species (ROS) within the cell. ROS production is usually low under physiological conditions, however, when pathological ROS production outpaces endogenous scavenging capacity; it can alter proteins and lipid function, triggering cell death, which typically occurs in heart failure, therefore, decreasing mitochondrial quality control and energy supply [16].

To decrease mitochondrial oxidative damage, many mitochondria-targeted antioxidants have been developed in the last few years and a promising mitochondrial antioxidant that has emerged is MitoQ, which consists of a quinone moiety linked to a triphenylphosphonium moiety by a 10-carbon alkyl chain [17].

MitoQ has been shown to protect against oxidative damage in many animal models of pathology, including cardiac ischemia–reperfusion (IR) injury [18], hypertension [19] but the effects on the development of heart failure remains understood. Therefore, our goal was to analyze the effects of MitoQ on the development and progression of heart failure induced by pressure overload.

## 2 Material and methods

### 2.1 Experimental design and aortic constriction surgery

The animal protocol was conducted according to the Guideline for the Care and Use of Laboratory Animals (NIH publication 85–23) and was approved by the University of Maryland School of Medicine Institutional Animal Care and Use Committee (Protocol number 1009011).

Animals were anesthetized with 2.5% isoflurane in oxygen, intubated and ventilated. A partial median sternotomy was performed and the thymus resected. After dissection of the aortic arch, a tantalum clip (.45 mm internal diameter-Hemoclip) was placed on the aorta between the brachiocephalic trunk and the left common carotid artery. The sternotomy was closed with interrupted sutures and the skin closed with running sutures. After their vital signs were reestablished, rats were extubated. Rats were kept in 100% oxygen and on warming pads during the surgery and recovery periods. Age-matched Sham-operated animals underwent the same procedure without clip application. Three days after surgery, the animals were assigned to receive MitoQ (100 µM in drinking water).

#### 2.1.1 Echocardiography

Cardiac function was assessed using a Vevo 2100 High-Resolution Imaging Systems (Visual Sonics) with a 15-MHz linear array transducer (MS200) at 13 weeks of treatment as previously described [20]. Rats were anesthetized with 2.5% isoflurane in oxygen, shaved and placed on a warming pad. Two-dimensional cine loops and guided M-mode frames were recorded from the parasternal short and long axes. At the end of the study, all data were analyzed offline with software resident on the ultrasound system, and calculations were made to determine left ventricular volumes. Ejection fraction was calculated as: (end-diastolic diameter ^__^ end-systolic diameter)/end-diastolic diameter. Relative wall thickness was calculated as the sum of the posterior and anterior wall thickness at end diastole divided by the end diastolic diameter. Mean wall thickness was taken as the average of the posterior and anterior wall thickness at end diastole. All calculations were made from parasternal short axis measurements.

#### 2.1.2 Tissue harvest

After 14 weeks of treatment, the animals were anaesthetized with 5.0% isoflurane between 3 and 6 h after initiation of the light phase while given free access to food. The thorax was opened and blood was collected from the left ventricle and immediately placed on ice tubes containing or not EDTA, and centrifuged at 1500 g for 15 min at 4 °C to obtain serum and plasma, respectively. The heart was removed, and three sections of the left ventricle free wall were taken for biochemical analysis and stored at −80 °C, and the remainder was used for mitochondrial isolation as described below.

#### 2.1.3 Mitochondrial isolation

Subsarcolemmal mitochondria (SSM) and interfibrillar mitochondria (IFM) were isolated as previously described [20], [21], [22]. Briefly, the LV was rinsed in ice cold Chappel-Perry buffer (100 mM KCl, 50 mM MOPS, 5 mM MgSO4, 1 mM ATP, 1 mM EGTA, 2 mg/mL BSA), blotted dry and then weighed. The ventricles were minced and homogenized in 1:10 (wt/vol) ice cold Chappel-Perry buffer. The homogenates were centrifuged at 580×*g* for 10 min. The supernatant containing SSM was extracted and centrifuged again at 5000×*g* to isolate SSM. The remaining pellet from the 580×*g* spin was resuspended in KCl-MOPS-EGTA buffer containing 100 mM KCl, 50 mM MOPS, and 0.5 mM EGTA at pH 7.4, and treated with trypsin (5 mg/g) for 10 min at 4 °C. The samples were incubated with Chappel-Perry buffer (albumin 2 mg/mL) to inhibit trypsin and spun down at 580×*g* for 10 min. The IFM-containing supernatant was spun down at 5000×*g* for 10 min. The pellets were washed in ice cold Chappel-Perry buffer and spun down at 5000×*g* for 10 min and washed in KME (100 mM KCl, 50 mM MOPS, and 0.5 mM EGTA at pH 7.4) and then resuspended in KME. The concentration of mitochondrial protein was measured by the Lowry method using bovine serum albumin as standard.

#### 2.1.4 Mitochondrial respiration

Mitochondrial respiration was assessed in both IFM and SSM as described previously [21], [23]. Isolated mitochondria (0.5 mg/mL) were respired in respiration buffer containing 100 mM KCl, 50 mM MOPS, 5 mM KH_2_PO_4_, 1 mM EGTA and 1 mg/mL BSA/Fraction V, using a polarographic oxygen sensing systems for measurements of dissolved oxygen consumption in liquid suspension (Qubit System, Kingston, ON, Canada). States 3 and 4 were measured with glutamate + malate (10 and 5 mM, respectively), palmitoylcarnitine (40 μM) and Succinate (20 mM) with rotenone (7.5 μM) was used to assess respiration through Complex II of the ETC exclusively. State 3 respiration (ADP-stimulated) was measured in the presence of 200 μM ADP. State 4 respiration (ADP-limited) was assessed after ADP consumption. Respiratory Control Ratio, the ratio of State 3 to State 4 was calculated to assess the control of oxygen consumption by phosphorylation. The ratio of ADP added in the chamber to the total amount of oxygen consumed in state 3 (ADP:O ratio) was calculated as an index of the efficiency of oxidative phosphorylation.

#### 2.1.5 Mitochondrial membrane potential (ΔΨm), size and complexity

Membrane potential, size and internal complexity were determined by flow cytometry analyses using Facscan (Becton Dickinson) as previously described [23]. Distinct mitochondrial subpopulations, IFM and SSM were incubated with the ratiometric dye 5,5′,6,6′-tetrachloro-1,1′,3,3′-tetraethylbenzimidazol carbocyanine iodide (JC-1; Invitrogen) for 15 min at 37 °C (final concentration 300 nM), which is a lipophilic cation that enters selectively into mitochondria, or MitoTracker Deep Red 633 (Invitrogen), which passively diffuses into intact mitochondria due to membrane potential and selectively stain intact mitochondria. Each individual parameter (gating, size, and complexity) was performed using specific light sources, detectors and 100,000 gated events were analyzed per sample. Measurements were performed on freshly isolated mitochondria and values are expressed as the mean orange fluorescence divided by the mean green fluorescence for membrane potential analyses (JC-1). For size and internal complexity analyses (MitoTracker), geometric mean representing FSC (forward scatter-logarithmic scale) was used as an indicator of size, whereas values from SSC (side scatter-logarithmic scale) were used to indicate complexity.

#### 2.1.6 Mitochondrial permeability transition pore (MPTP) opening assay

MPTP opening in SSM and IFM was performed as previously described [20], [24]. Brief, mitochondria (500 µg) were resuspended in 2.0 mL assay medium [containing 100 mM KCl, 50 mM MOPS, 5 mM KH_2_PO_4_, 5 mM EGTA, 1 mM MgCl_2_, 5 mM glutamate, and 5 mM malate] and assayed for tert-butyl-induced pore opening in a fluorescence spectrophotometer at 37 °C. Calcium uptake was taken from the fall in extramitochondrial Ca^2+^ following a bolus injection of 3 μL of 15 mM Ca^2+^ (30 nmoles Ca^2+^/mg mitochondrial protein) and tert-butyl-induced calcium release were assessed in both isolated subpopulations SSM and IFM after calcium uptake. Tert-butyl hydrogen peroxide (400 mM) was infused at a rate of 0.2 μL/min, and the concentration of free Ca^2+^ in the medium was calculated by monitoring the fluorescence of the Ca^2+^ indicator calcium green-5N (CaGN-5N; Molecular Probes) with an excitation and emission of 488 and 530, respectively. MPTP opening was inferred from the sudden and large increase of fluorescence.

#### 2.1.7 Swelling assay

Ca^2+^ tolerance was evaluated according to previous reports [21], [25], [26]. Briefly, mitochondria were resuspended in swelling buffer [containing 100 mM KCl, 50 mM MOPS, 5 mM KH_2_PO_4_, 5 µM EGTA, 1 mM MgCl_2_, 5 mM glutamate, and 5 mM malate] at 2 mg/mL protein concentration and warmed to room temperature in a 96-well plate. CaCl_2_ (100 and 500 nM/mg of mitochondrial protein) was added. The time-dependent decrease in absorbance at 540 nm is indicative of the tendency for MPTP opening and mitochondrial swelling.

#### 2.1.8 Hydrogen peroxide production

Hydrogen peroxide production in isolated mitochondrial subpopulations was determined using the oxidation of the fluorogenic indicator Amplex Red (Invitrogen) in the presence of horseradish peroxidase as previously described [21]. The concentrations of horseradish peroxidase and Amplex Red in the incubation were 0.1 unit/mL and 50 μM, respectively, and detection of fluorescence was assessed on a on a Molecular Devices Flex Station 3 fluorescence plate reader (Molecular Devices, Sunnyvale, CA) with 530-nm excitation and 590-nm emission wavelengths. Standard curves were obtained by adding known amounts of H_2_O_2_ to the assay medium in the presence of the substrates amplex red and horseradish peroxidase. H_2_O_2_ production was initiated in mitochondria using glutamate + malate (G + M) and succinate + rotenone (S + R) as substrates.

#### 2.1.9 Enzyme activity

Citrate synthase, MCAD and aconitase activities were measured as previously described [27]. For citrate synthase, briefly, 0.5 μL of myocardial tissue homogenate was added to cuvettes containing 500 μL of CS reaction mix (0.1 M Tris-HCl, 1.25 mM 5,5′-dithiobis [2-nitrobenzoic acid], pH 8). Then, 25 μL of 50 mM oxaloacetate and 5 mM acetyl-CoA were added to the reaction mixture, and the increase in absorbance at 412 nm was measured over 5 min. Activity was then determined by multiplying the slope to the molar concentration constant for 5,5′-dithiobis[2-nitrobenzoic acid] (13,600 M^−1^).

For MCAD activity, 5 μL of myocardial tissue homogenate was added to cuvettes containing 500 μL of 100 mM KH_2_PO_4_, 1 mM EDTA, 0.5 mM sodium tetrathionate, and 200 μM ferrocenium hexafluorophosphate, pH 7.4. Then, 50 μL of 0.5 mM octanoyl-CoA was added to the reaction mixture, and the decrease in absorbance at 300 nm was measured over 5 min. Activity was then determined by multiplying the slope to the molar concentration constant for ferrocenium hexafluorophosphate (4300 M^−1^).

For aconitase activity, 10 μL of myocardial tissue homogenate was added to cuvettes containing 490 μL of aconitase reaction mix (0.083% chloroform, 1.67 mM sodium citrate, 26.7 mM triethanolamine, 0.5 mM NADP^+^, .5 mM MgCl_2_, pH 7.4), and the increase in absorbance at 340 nm was measured over 5 min. Activity was then determined by multiplying the slope to the molar concentration constant for NADPH (6.7 M^−1^).

## 3 Statistical analysis

Values are shown as mean ± standard error of the mean (SEM) unless otherwise specified. For comparisons between groups, we used one or two-way analysis of variance (1 or 2-way ANOVA), and if statistically significant differences were detected, Fischer's post hoc test was applied to further identify groups with different means. Differences were considered significant for P values of less than 0.05.

## 4 Results

### 4.1 Development of heart failure in response to pressure overload

Heart Failure became manifest at 14 weeks following aortic constriction, as indicated by a 50% increase in LV mass, hypertrophy of the atria and right ventricle, greater LV all thickness, significant increases in LV end systolic and diastolic volumes and a decreased ejection fraction. Furthermore, MitoQ decreased right ventricular hypertrophy and lung congestion induced by pressure overload after 14 weeks. Although TAC animals did not present ejection fraction below 30–40% which is the main criteria for heart failure classification, the animals present Lung/BW ≥ Lung/BW_Sham_; + 2 SD (Lung wet weight; BW = body weight; SD = standard deviation); RV hypertrophy was proportional to Lung/BW (Table 1, Table 2) [26], [28].

**Table 1:** MitoQ does not alter cardiac hypertrophy in the left ventricle induced by transverse aortic constriction but improves hypertrophy in the right ventricle after 14 weeks of treatment. Changes in body weight (BW), left ventricle to tibia length ratio (LV/tibia), right ventricle weight to tibia length ratio (RV/tibia), atrial weight to tibia length ratio (AW/tibia), liver weight, retroperitoneal + epididymal fat (% of total weight), lung (wet to dry ratio), kidney, gastrocnemius, soleus in SHAM, TAC and TAC plus MitoQ. N - Number of animals used. *p < .05 compared to SHAM. #p < .05 compared to TAC. Results are expressed as the means ± SEM. Differences were analyzed using one-way ANOVA followed by a post hoc Fischer test.

	SHAM (N = 13)	TAC (N = 15)	TAC MitoQ (N = 15)
**Body weight (g)**	414 ± 8	390 ± 6.7	403 ± 6.9
**LV/tibia (mg/mm)**	22 ± 0.42	33 ± 0.76*	34 ± 1.2*
**RV/tibia (mg/mm)**	5 ± 0.16	10.5 ± 0.29*	8.9 ± 0.501*, #
**AW/tibia (mg/mm)**	1.6 ± 0.09	5.4 ± 0.33*	4.8 ± 0.33*
**Liver (g)**	12 ± 0.23	13 ± 0.27	13 ± 0.33
**Retroperitoneal + epididymal fat (% of total weight)**	20 ± 1.3	14 ± 0.7*	15 ± 0.63*
**Lung weight (g)**	1.47 ± 0.05	5.23 ± 0.18*	4.23 ± 0.44*, #

Data are means ± SEM. LV/tibia, left ventricle-to-tibia ratio; RV/tibia, right ventricle-to-tibia ratio; AW/tibia, atrial weigh-to-tibia ratio.

* P < .05 vs SHAM.

# P < .05 vs TAC. Differences were analyzed using one-way ANOVA followed by a post hoc Fischer test.

**Table 2:** MitoQ does not change echocardiography indices of transverse aortic constriction induced HF model in rats after 14 weeks of treatment. Changes in left ventricular end diastolic diameter, left ventricular end systolic diameter, end diastolic anterior wall thickness, end diastolic posterior wall thickness, endocardial fractional area change (%), diastolic volume, systolic volume, left ventricular fractional shortening and ejection fraction (%) in SHAM, TAC and TAC plus MitoQ. N - Number of animals used. *p < .05 compared to SHAM. Results are expressed as the means ± SEM. Differences were analyzed using one-way ANOVA followed by a post hoc Fischer test.

	**SHAM (N = 13)**	**TAC (N = 15)**	**TAC MitoQ (N = 15)**
**LV end diastolic diameter (mm)**	7.7 ± 0.17	8.4 ± 0.22	8.3 ± 0.26
**LV end systolic diameter (mm)**	4.3 ± 0.16	5.9 ± 0.19*	5.7 ± 0.29
**End diastolic anterior wall thickness (mm)**	1.9 ± 0.096	2.9 ± 0.11*	3.1 ± 0.12*
**End diastolic posterior wall thickness (mm)**	2.1 ± 0.14	2.7 ± 0.11*	2.8 ± 0.15*
**Endocardial Fractional Area Change (%)**	64 ± 2.5	42 ± 2.3*	41 ± 3.2*
**Diastolic Volume (mL)**	0.49 ± 0.033	0.64 ± 0.053*	0.62 ± 0.056*
**Systolic Volume (mL)**	0.089 ± 0.0092	0.23 ± 0.023*	0.22 ± 0.029*
**LV fractional shortening**	0.44 ± 0.016	0.29 ± 0.015*	0.31 ± 0.021*
**Ejection Fraction (%)**	82 ± 1.4	64 ± 2.3*	66 ± 2.7*

Data are means ± SEM.

* P < .05 vs SHAM. Differences were analyzed using one-way ANOVA followed by a post hoc Fischer test.

### 4.2 Mitochondrial yield, membrane potential and morphology

**Fig. 1:**
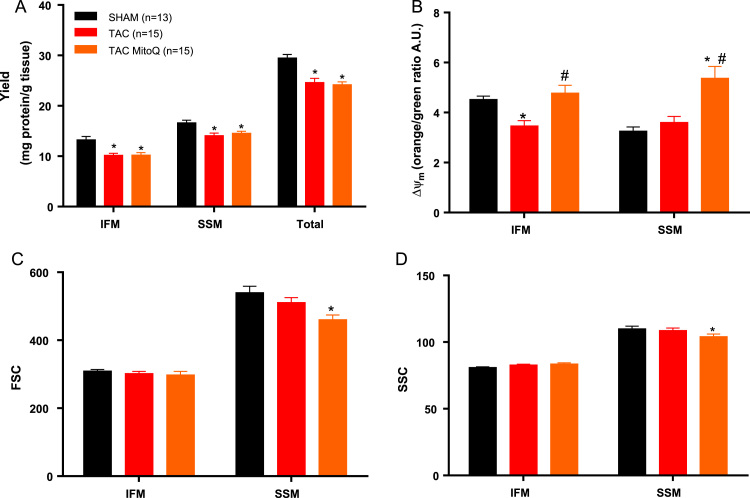
**MitoQ restores mitochondrial membrane potential, decreases size and internal complexity in subsarcolemmal mitochondria in heart failure after 14 weeks of transverse aortic constriction**. Changes in mitochondrial yield, membrane potential, size and internal complexity in SHAM, TAC and TAC plus MitoQ. N - Number of animals used. *p < .05 compared to SHAM. #p < .05 compared to TAC. Results are expressed as the means ± SEM. Differences were analyzed using one-way ANOVA followed by a post hoc Fischer test. (For interpretation of the references to color in this figure, the reader is referred to the web version of this article.)

The heart failure group had a significant 23% decrease in IFM yield and a 16% decrease in SSM yield that were not altered by MitoQ (mg of mitochondrial protein extracted per gram of myocardium) (Fig. 1A). Thus, the heart failure group had a lower total mitochondrial content in myocardium. Further, we also measured mitochondrial membrane potential by flow cytometry. For membrane potential, mitochondria were incubated with 5,5′,6,6′-tetrachloro-1,1′,3,3′-tetraethylbenzimidazol carbocyanine iodide (JC-1) at a final concentration of 0.3 μM. The shift to orange is due to dye aggregates forming upon polarization, causing shifts in emitted light from 530 nm (green) to 590 nm (orange). The membrane potential was decreased in the IFM from the heart failure group. Interestingly, MitoQ restored membrane potential in the IFM. MitoQ also increased the membrane potential in the SSM, although the membrane potential was not found reduced in the TAC group for this subpopulation (Fig. 1B).

To assess mitochondrial morphology and internal complexity, the arithmetic mean output from the forward scatter (FSC) detector was used as an index of mitochondrial size and the arithmetic mean output from the side scatter was used as an index of mitochondrial internal complexity. Size and internal complexity were not altered in both subpopulation in the heart failure group. However, MitoQ reduced size and internal complexity in heart failure (Fig. 1C and D).

### 4.3 Mitochondrial respiration

**Fig. 2:**
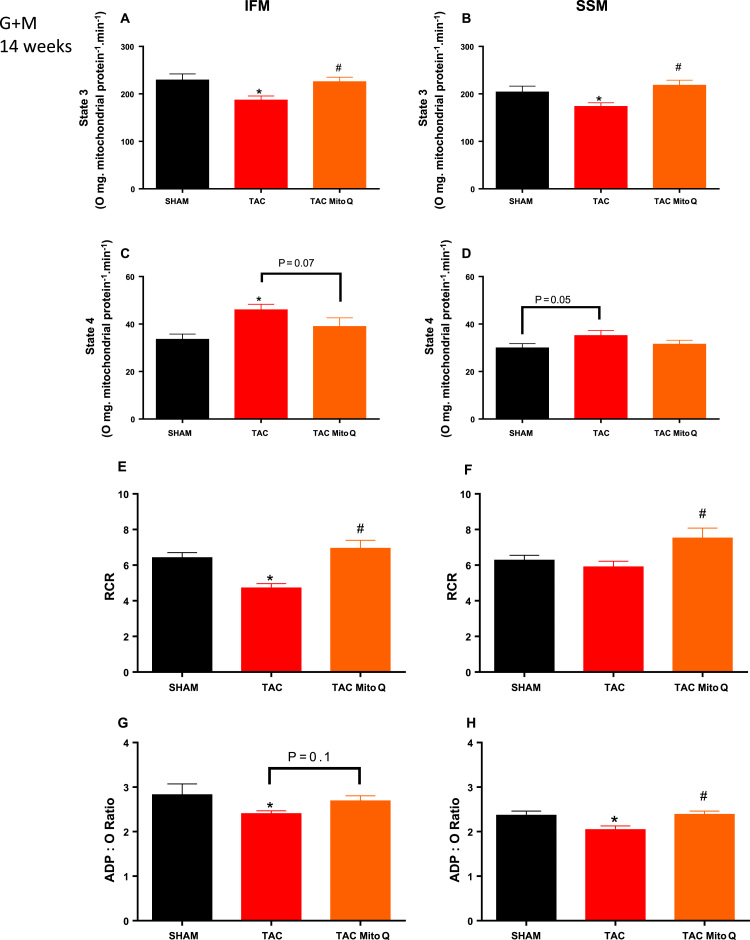
**MitoQ restores mitochondrial dysfunction in IFM and SSM in heart failure induced by pressure overload after 14 weeks of transverse aortic constriction**. Mitochondrial subpopulations respiration. Individual mitochondrial subpopulations were isolated from SHAM, TAC and TAC plus MitoQ hearts, and polarographic measurements were performed to index oxygen consumption under state 3 and 4 respiration conditions. Respiration of individual electron transport chain (ETC) complex was defined as the rate of oxygen consumed in the presence of specific substrate (glutamate and malate) for complex I. All respiration rates are expressed in Atoms O.mg mitochondrial protein^-1^ min^−1^. RCR (respiratory control ratio, state 3/state 4) and the ratio of ADP:O in isolated subsarcolemmal and Intermyofibrillar mitochondria. Number of animals used 13–15 per group. *p < .05 compared to SHAM. #p < .05 compared to TAC. Results are expressed as the means ± SEM. Differences were analyzed using one-way ANOVA followed by a post hoc Fischer test.

To study the effect of heart failure on different functional components of IFM and SSM, we measured mitochondrial respiration with different substrates (palmitoylcarnitine, glutamate + malate, and succinate) that use distinct mitochondrial transport, oxidative pathways and provide specific substrates to Complex I and Complex II. To assess β-oxidation, we used palmitoylcarnitine that reflects mitochondrial palmitoylcarnitine transport, palmitate oxidation, the activity of entire ETC and the phosphorylation process. The combination of glutamate and malate as substrate produces NADH, NADH donates electrons for Complex I, thus allowing the study of the aspartate shuttle. Succinate is oxidized in Complex II (bypassing Complex I) and the addition of rotenone, an inhibitor of Complex I, allows assessing the activity of complex II. The maximal rate of mitochondrial respiration (state 3) in isolated mitochondria expressed per mg of mitochondrial protein was decreased by 20% in IFM and 16% in SSM from heart failure animals with glutamate + malate as substrates, furthermore, MitoQ restored mitochondrial respiration (state 3) in both subpopulations (Fig. 2A and 2B). We also detected an increase in state 4 in IFM from heart failure rats with glutamate + malate as substrates. Although MitoQ did not reduce the increase in state 4 in IFM from heart failure animals, there was a tendency to be statistically significant (P = .07) (Fig. 2C). On the other hand, state 4 was not different in SSM but there was a tendency to be increased in heart failure animals (P = .05). In addition, heart failure animals treated with MitoQ showed similar values for state 4 when compared to the SHAM group (Fig. 2D).

The respiratory control ratio (state 3/state 4), an index of the ability of mitochondria to increase ATP generation above basal values in response to maximally stimulating conditions, was decreased in IFM but not SSM in heart failure when glutamate + malate as substrates. MitoQ restored the decrease in RCR in IFM (Fig. 2E) and, although the RCR in SSM from heart failure animals was not decreased, heart failures animals treated with MitoQ showed higher values when compared to the heart failure group (Fig. 2F). ADP/O ratio was also found decreased in IFM and SSM from heart failure animals. In addition, MitoQ restored the ADP/O ration in SSM but not in IFM (Figs. 2G and 2H).

**Fig. 3:**
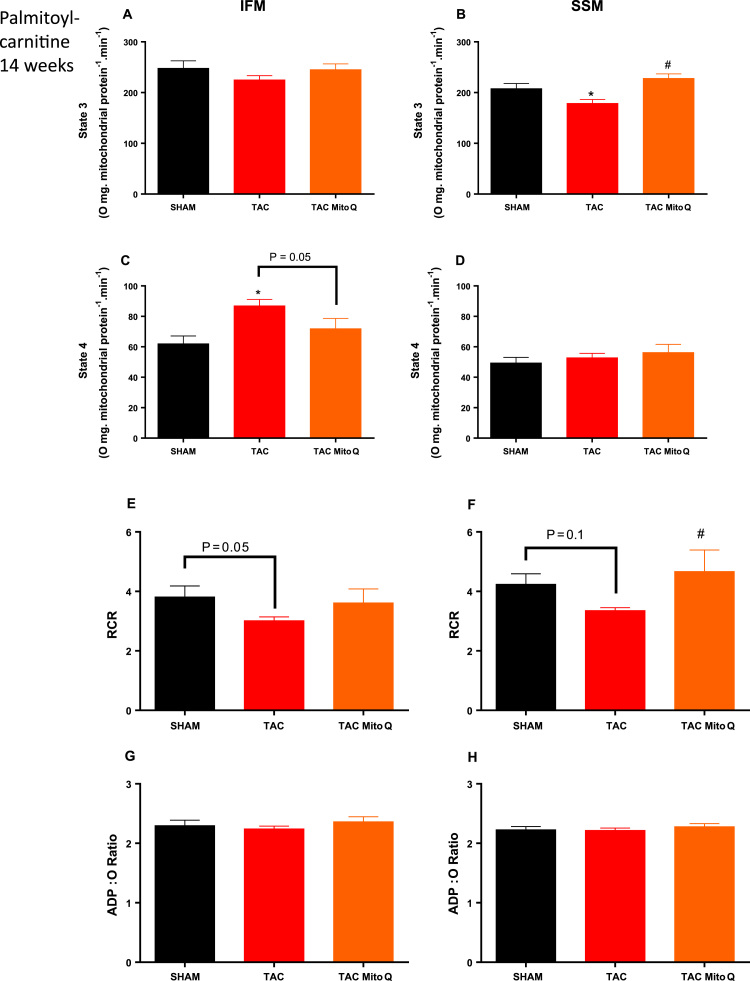
**MitoQ restores β-oxidation in subsarcolemmal mitochondria from heart failure induced by pressure overload after 14 weeks of transverse aortic constriction**. Mitochondrial subpopulations respiration. Individual mitochondrial subpopulations were isolated from SHAM, TAC and TAC plus MitoQ hearts, and polarographic measurements were performed to index oxygen consumption under state 3 and 4 respiration conditions. Respiration of individual electron transport chain (ETC) complex was defined as the rate of oxygen consumed in the presence of specific substrate (Palmitoyl-carnitine) for β-oxidation. All respiration rates are expressed in Atoms O.mg mitochondrial protein^-1^ min^−1^, RCR (respiratory control ratio, state 3/state 4) and the ratio of ADP:O in isolated subsarcolemmal and Intermyofibrillar mitochondria. Number of animals used 13–15 per group. *p < .05 compared to SHAM. #p < .05 compared to TAC. Results are expressed as the means ± SEM. Differences were analyzed using one-way ANOVA followed by a post hoc Fischer test.

We next aimed to analyze β-oxidation and to doing that, we used palmitoylcarnitine, which reflects mitochondrial palmitoylcarnitine transport and palmitate oxidation. As expected, the maximal rate of mitochondrial respiration (state 3) in isolated mitochondria expressed per mg of mitochondrial protein was decreased in SSM but not IFM from heart failure rats. MitoQ restored this decrease found in heart failure animals (Fig. 3A and B). We also detected higher values for state 4 in IFM but not SSM from heart failure animals when palmitoylcarnitine was used as substrate. Furthermore, MitoQ restored the increase in state 4 in IFM from heart failure animals (Fig. 3C and D).

The respiratory control ratio (state 3/state 4) was reduced in IFM and SSM from heart failure animals, although there was a tendency to be statistically significant only in IFM (P = .05). Interestingly, heart failure animals treated with MitoQ showed similar values compared to the SHAM group (Fig. 3E and F). The ADP/O ratio not different among groups when palmitoylcarnitine was used as substrate (Fig. 3G and H).

**Fig. 4:**
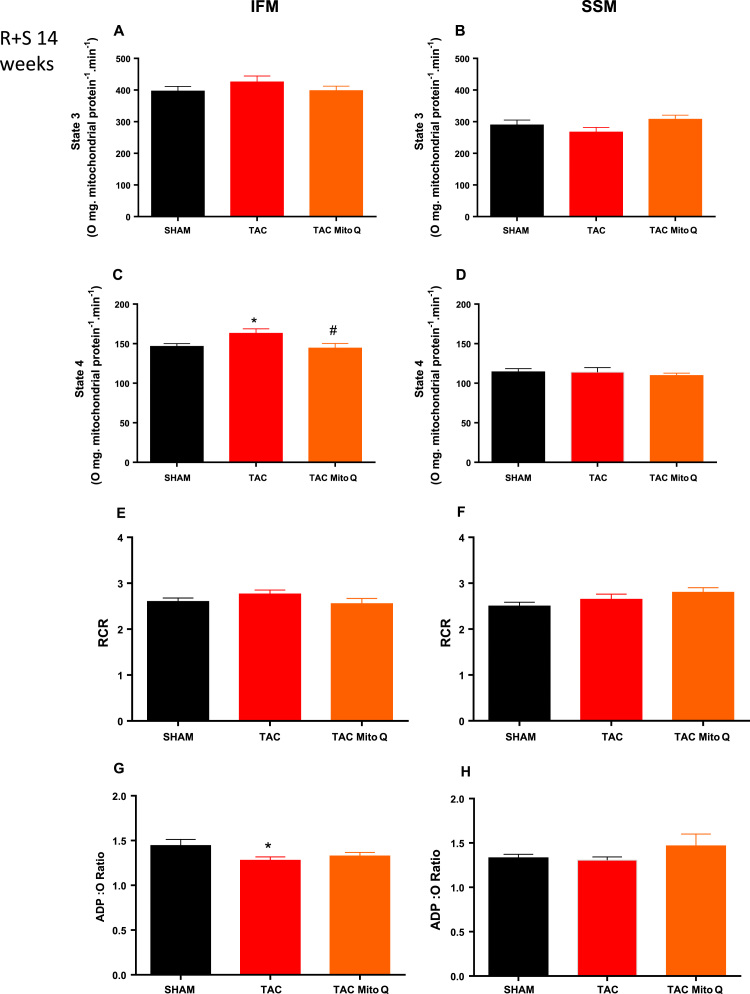
**MitoQ restores the increase in state 4 in intermyofibrillar mitochondria from heart failure induced by pressure overload after 14 weeks of transverse aortic constriction**. Mitochondrial subpopulations respiration. Individual mitochondrial subpopulations were isolated from SHAM, TAC and TAC plus MitoQ hearts, and polarographic measurements were performed to index oxygen consumption under state 3 and 4 respiration conditions. Respiration of individual electron transport chain (ETC) complex was defined as the rate of oxygen consumed in the presence of specific substrate (succinate + rotenone) for complex II. All respiration rates are expressed in Atoms O.mg mitochondrial protein^-1^ min^−1^, RCR (respiratory control ratio, state 3/state 4) and the ratio of ADP:O in isolated subsarcolemmal and Intermyofibrillar mitochondria. Number of animals used 13–15 per group. *p < .05 compared to SHAM. #p < .05 compared to TAC. Results are expressed as the means ± SEM. Differences were analyzed using one-way ANOVA followed by a post hoc Fischer test.

Finally, we inhibited complex I with rotenone and provided substrate for complex II (succinate). Heart failure did not affect state 3 with succinate as substrate in both subpopulations (Fig. 4A and B). Furthermore, state 4 was found increased in the IFM but not in SSM (Fig. 4C and D). We also analyzed RCR and ADP:O ratio in both subpopulations and we found a modest decrease in the ADP:O ratio in IFM but not SSM (Fig. 4E, F, G and H).

Taken together, heart failure resulted in loss of mitochondrial content and tissue respiratory capacity in both subpopulations and MitoQ was able to improve respiratory capacity.

### 4.4 Effects of heart failure on biochemical parameters

**Fig. 5:**
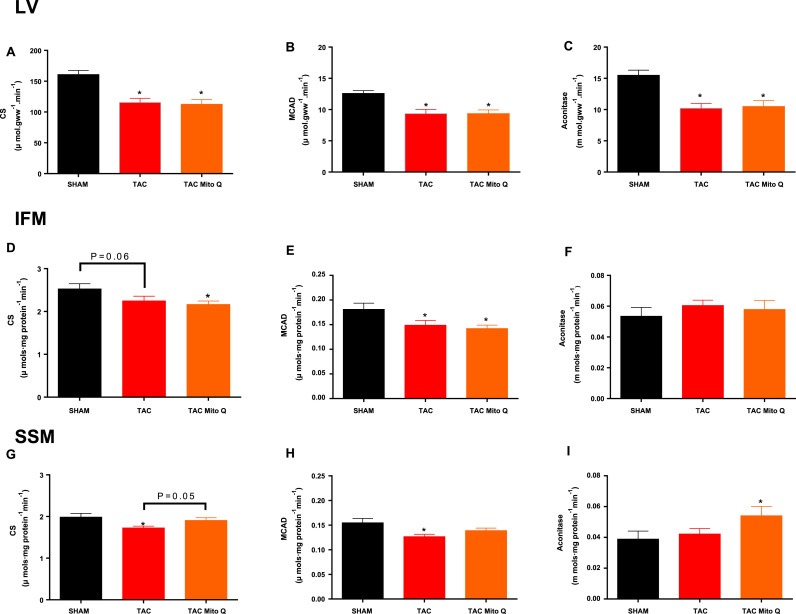
**Heart failure decreases myocardial activity of mitochondrial function and the fatty acid oxidation pathway**. Myocardial activity of citrate synthase (CS), medium chain acyl-CoA dehydrogenase (MCAD) (B) and aconitase (C). Activity of citrate synthase (CS) (D), medium chain acyl-CoA dehydrogenase (MCAD) (E) and aconitase (F) in the intermyofibrillar mitochondria. Activity of citrate synthase (CS) (G), medium chain acyl-CoA dehydrogenase (MCAD) (H) and aconitase (I) in the subsarcolemmal mitochondria. Individual mitochondrial subpopulations were isolated from SHAM, TAC and TAC plus MitoQ hearts. Number of animals used 13–15 per group. *p < .05 compared to SHAM. #p < .05 compared to TAC. Results are expressed as the means ± SEM. Differences were analyzed using one-way ANOVA followed by a post hoc Fischer test.

Heart failure decreases the capacity for oxidative metabolism in myocardium, as reflected in a decrease in the activities of citrate synthase, aconitase and medium-chain acyl-CoA dehydrogenase (MCAD). The activities of these enzymes were decreased by pressure overload-induced heart failure and were not affected by MitoQ treatment (Fig. 5A, B and C).

We also measured the activities of these enzymes in both subpopulations separately and we found that citrate synthase and MCAD were decreased by heart failure-induced by pressure overload in IFM and SSM (Fig. 5D, E, G and H) whereas aconitase as not altered by hear failure in the isolated mitochondria (Fig. 5F and I). Furthermore, MitoQ did not alter the decrease in mitochondrial enzyme activity, however, aconitase activity in the SSM was found increased in heart failure group treated with MitoQ (Fig. 5I).

### 4.5 Hydrogen peroxide production

**Fig. 6:**
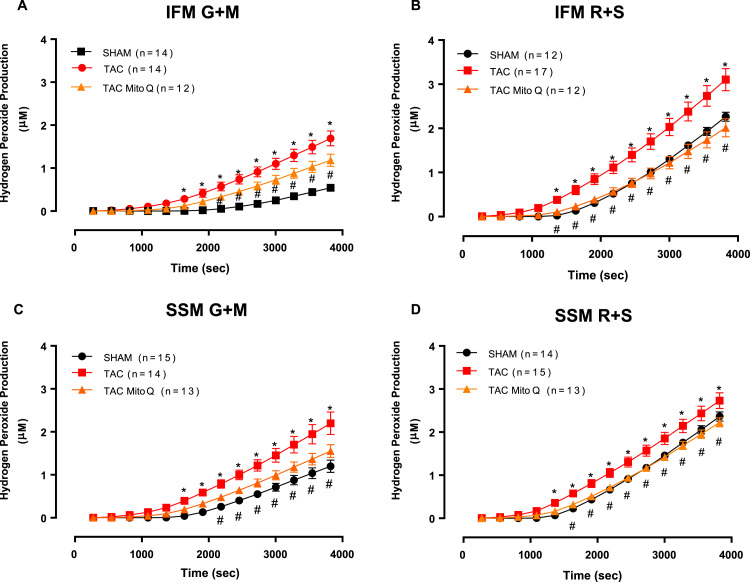
**MitoQ restores hydrogen peroxide production in intermyofibrillar and subsarcolemmal mitochondria from heart failure induced by pressure overload after 14 weeks of transverse aortic constriction**. Hydrogen peroxide production in intermyofibrillar and subsarcolemmal mitochondria from individual mitochondrial subpopulations isolated from SHAM, TAC and TAC plus MitoQ hearts. Two different substrates were used to assess hydrogen peroxide production from complex I (glutamate + malate) and complex III (rotenone + succinate). Number of animals used is indicated in parenthesis. *p < .05 compared to SHAM. #p < .05 compared to TAC. Results are expressed as the means ± SEM. Differences were analyzed using two-way ANOVA followed by a post hoc Fischer test.

The capacity for mitochondrial generation of H_2_O_2_ was assessed in IFM and SSM from LV myocardium using the amplex red assay. Heart failure significantly increased mitochondrial generation of H_2_O_2_ in both subpopulations, IFM and SSM with either glutamate + malate or succinate in the presence of the rotenone to inhibit complex I. As expected, MitoQ normalized hydrogen peroxide production in heart failure-induced by pressure overload (Fig. 6A, B, C and D).

### 4.6 Permeability transition

**Fig. 7:**
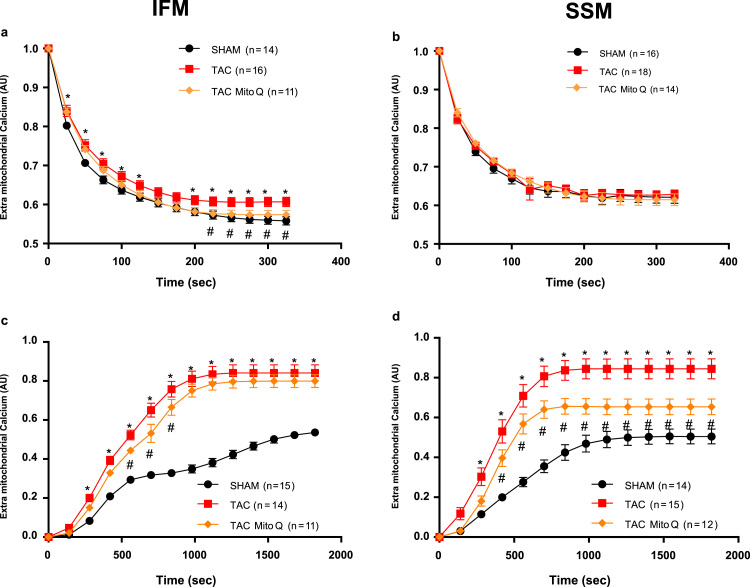
**Calcium-uptake capacity and reactive oxygen species (ROS) sensitivity of mitochondria isolated from SHAM, TAC and TAC plus MitoQ hearts**. A: rate of Ca^2+^ uptake by interfibrillar mitochondria (IFM) is monitored as a decrease in fluorescence of the extra-mitochondrial Ca^2+^ indicator (CaGN-5N; 700 nM). B: decrease in CaGN-5N fluorescence over time due to the uptake of Ca^2+^ by subsarcolemmal mitochondria (SSM). C: IFM mitochondria were primed for mitochondrial permeability transition pore (MPTP) opening with a low concentration of Ca^2+^ (22.5 µM) and were subsequently exposed to increasingly higher doses of tBH. Mitochondrial Ca^2+^ release as a result of tBH-induced MPTP was monitored as an increase in CaGN-5N fluorescence. D: increase in CaGN-5N fluorescence due to tBH-induced MPTP opening in SSM. AU, arbitrary fluorescence units; tBH, tert-butyl-hydroxyperoxide.). Number of animals used is indicated in parenthesis. *p < .05 compared to SHAM. #p < .05 compared to TAC. Results are expressed as the means ± SEM. Differences were analyzed using two-way ANOVA followed by a post hoc Fischer test.

Two established methods were used to assess Ca^2+^-induced MPT in LV mitochondria. First, it was assessed from the measurement of the ability of isolated mitochondria to take up added Ca^2+^. A small amount of calcium was added to the medium (30 nmoles Ca^2+^/mg mitochondrial protein) and the fluorescence was measured over 5 min. After stabilization, tert-butyl hydrogen peroxide (400 mM) was infused at a rate of 0.2 μL/min, and the concentration of free Ca^2+^ in the medium was calculated by monitoring the fluorescence of the Ca^2+^ indicator calcium green-5N. Heart failure decreased the ability of IFM to take up calcium whereas SSM did not show any difference among groups. MitoQ was able to improve the maximal rate of mitochondrial calcium uptake in IFM (Fig. 7A).

There was a significantly greater sensitivity to tert-butyl hydrogen peroxide-induced MPT in IFM and SSM in heart failure group as reflected by a higher extramitochondrial [Ca^2+^] for a given cumulative load of tert-butyl hydrogen peroxide. MitoQ was able to improve tert-butyl hydrogen peroxide-induced MPT in IFM and SSM (Fig. 7C and D).

**Fig. 8:**
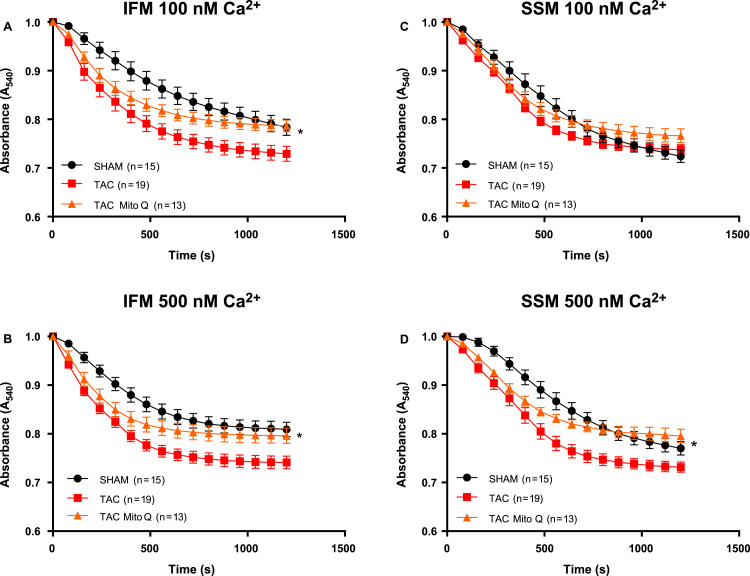
**MitoQ improves calcium-induced mitochondrial swelling in intermyofibrillar and subsarcolemmal mitochondria from heart failure induced by pressure overload after 14 weeks of transverse aortic constriction**. (A) Total cardiac mitochondria were isolated from SHAM, TAC and TAC plus MitoQ hearts and exposed to 100 and 500 nM Ca^2+^/mg mitochondrial protein to induce swelling. The change in absorbance that occurs as a result of mitochondrial swelling is monitored over time at 540 nm and is expressed relative to the absorbance at the beginning of the assay. Number of animals used is indicated in parenthesis. *p < .05 compared to SHAM. #p < .05 compared to TAC. Results are expressed as the means ± SEM. Differences were analyzed using two-way ANOVA followed by a post hoc Fischer test.

Second, MPT was assessed from mitochondrial swelling induced by high [Ca^2+^] as reflected by the decrease in absorbance at 540 nm following the addition of a bolus of Ca^2+^ to isolated mitochondria. There was a decrease in absorbance with addition of either 100 or 500 nmol Ca^2+^/mg protein in all groups, with a greater decline in IFM and SSM from heart failure-induced by pressure overload. MitoQ treatment improved the ability of isolated mitochondria to swell in IFM and SSM with addition of either 100 or 500 nmol Ca^2+^/mg (Fig. 8A, B, C and D).

## 5 Discussion

This study was designed to assess the effect of MitoQ on pressure overload-induced heart failure in two diverse mitochondrial populations, IFM and SSM, characterized with distinct biochemical properties and functions. The main findings are that pressure overload-induced heart failure in rats decreased cardiac function *in vivo* that was not altered by MitoQ. However, we observed a slight improvement in RV hypertrophy in heart failure animals treated with MitoQ for 14 weeks. Heart failure also decreased total mitochondrial protein content, mitochondrial membrane potential in the intermyofibrillar mitochondria. MitoQ restored membrane potential in IFM but did not alter mitochondrial protein content. These alterations are associated with the impairment of basal and stimulated mitochondrial respiration in IFM and SSM induced by heart failure. Moreover, MitoQ restored mitochondrial respiration in heart failure induced by pressure overload. We also detected higher levels of hydrogen peroxide production in heart failure and MitoQ restored the increase in ROS production after 14 weeks. MitoQ was also able to increase mitochondrial calcium retention capacity.

Epidemiological studies show that cardiac hypertrophy is a strong predictor for the development of heart failure and it has been postulated that the reduction in oxidative phosphorylation accelerates the decline during the transition from left ventricular hypertrophy to heart failure as mentioned in the introduction.

Heart failure induced by pressure overload differently affects the two distinct mitochondrial subpopulations. As described in the literature, IFM is smaller and shows reduced internal complexity than SSM. Our data also shows higher respiratory capacity in IFM than in SSM. Heart failure after 14 weeks decreased mitochondrial respiratory capacity, thus our findings are consistent with previous results [29], [30]. This information is relevant because differences between IFM and SSM are often not addressed in recent publications, these two distinct subpopulations respond to pathological conditions in a heterogeneous fashion, such as diabetes [31], in cardiomyopathic hamsters [32] and heart failure [33], [34]. Additionally, we describe for the first time that MitoQ restored mitochondrial stimulated and limited respiration after 14 weeks in heart failure. This result corroborates previous findings showing that mice pre-treated with MitoQ had enhanced recovery after ischemia/reperfusion injury, demonstrating that MitoQ inhibited accumulation of reactive oxygen species and apoptosis induction [35].

It is well known that the failing heart has reduced capacity for oxidizing fatty acid as fuel. Down regulation of fatty acid oxidation gene expression is a well-characterized response in the hypertrophied myocardium driven, at least in part, by reduced PPAR alpha [5], [36], [37], [38], [39], which is responsible for transcriptional control of genes involved in fatty acid utilization [40]. To confirm this hypothesis, we used palmitoylcarnitine to evaluate palmitate transport and oxidation. Our findings show that β-oxidation is reduced in heart failure due to a decrease in state 3 respiration with palmitoylcarnitine as substrate in the SSM. Interestingly, MitoQ restored stimulated respiration with this substrate. We also demonstrated that respiratory control ratio is decreased in heart failure. Mitochondrial respiratory control ratio encapsulates the main function of mitochondria, which is the ability to idle at a low rate yet respond to ADP by making ATP at a high rate. Moreover, MitoQ normalized RCR values when compared to the heart failure group.

To confirm this result, MCAD activity was found decreased in the LV, IFM and SSM in heart failure, although MitoQ did not restore the activity of this enzyme, the activity of MCAD in SSM from heart failure group treated with MitoQ was not reduced as compared to SHAM. These results suggest that MitoQ could be useful to prevent the substrate shift found in heart failure. It has been shown that overexpression of mitochondrial catalase attenuates pressure overload-induced heart failure, demonstrating that scavenging mitochondrial ROS preserves the fatty acid oxidation pathway in heart failure [41].

Mitochondria has been designated as the main source of reactive oxygen species within the cardiomyocyte. Excessive ROS production from mitochondria has been described in heart failure [42], [43], leading to permeability and irreversible injury of the mitochondria [44]. Considering these data, direct targeting mitochondrial ROS by agents which accumulate in mitochondria such as MitoQ, have emerged as promising agents. MitoQ is oxidized by ROS and is reduced by electrons from complex II [45], [46]. Our data shows that mitochondrial hydrogen peroxide was normalized by MitoQ treatment after 14 weeks, demonstrating that MitoQ was efficient in reducing mitochondrial ROS production in heart failure.

ROS and calcium also favor permeability transition pore opening in mitochondria. ROS, including H_2_O_2_ can greatly sensitize the mPTP to Ca^2+^ and triggers mitochondrial permeability transition [47]. Our data shows that MitoQ decreased mitochondrial permeability in a greater extension in SSM but we observed a slight change in IFM, demonstrating that decreasing mitochondrial ROS production can reduce mPTP formation. We cannot rule out the possibility that MitoQ is effective in SSM due to its location within the cell. The limitations of the present investigation needs to be addressed. MitoQ did not alter LV hypertrophy or function *in vivo*, however, we observed a decrease in RV and lung weight as compared to the sham group, demonstrating that MitoQ had some significant effect on these organs. Regarding ventricular function, it is very well known that its function depends on preload, afterload and heart rate. In this *in vivo* model with changes in diastolic volume and severe hypertrophy, it is not adequate to conclude that MitoQ had no beneficial effects on ventricular function. Further experiments such as using isolated heart will be necessary to investigate whether MitoQ affects ventricular function. Furthermore, in TAC models, collagen deposition and fibrosis is present and we cannot rule out that MitoQ might decrease collagen deposition in this model but further experiments will be necessary to address this issue.

The difference that was not found in the LV could be attributed to the small concentration used in drinking water (100 µM). To confirm this hypothesis, a higher concentration must be used to treat HF animals.

## 6 Conclusion

In summary, MitoQ improves mitochondrial dysfunction in heart failure induced by pressure overload, by decreasing hydrogen peroxide formation, improving mitochondrial respiration and improving mPTP formation.

## Conflict of interest

The authors declare that there are no conflicts of interest related to this work.

## Sources of funding

This study was supported by grants from CNPq (455294/2014-3) and by the National Institutes of Health, Grant numbers HL074237, HL101434 and HL072751. Work in MPM’s lab is supported by the Medical Research Council UK (MC_U105663142) and by a Wellcome Trust Investigator award to MPM (110159/Z/15/Z) The funders had no role in study design, data collection and analysis, decision to publish, or preparation of the manuscript.

## Author contribution

Rogério Faustino Ribeiro Júnior, Erinne Dabkowski, Kadambari Chandra Shekar, Kelly A. O´Connell, Peter A Hecker performed the experiments, analyzed the data, discussed the results and wrote the paper, Michael Murphy was involved in discussing the results and writing the paper.
